# Chronic Cladribine Administration Increases Amyloid Beta Peptide Generation and Plaque Burden in Mice

**DOI:** 10.1371/journal.pone.0045841

**Published:** 2012-10-03

**Authors:** Crystal D. Hayes, Debleena Dey, Juan Pablo Palavicini, Hongjie Wang, Wataru Araki, Madepalli K. Lakshmana

**Affiliations:** 1 Section of Neurobiology, Torrey Pines Institute for Molecular Studies, Port Saint Lucie, Florida, United States of America; 2 Department of Demyelinating Disease and Aging, National Institute of Neuroscience, National Center for Neurology and Psychiatry (NCNP), Kodaira, Tokyo, Japan; Massachusetts General Hospital, United States of America

## Abstract

**Background:**

The clinical uses of 2-chloro-2′-deoxyadenosine (2-CDA) or cladribine which was initially prescribed to patients with hematological and lymphoid cancers is now extended to treat patients with multiple sclerosis (MS). Previous data has shown that 2-CDA has high affinity to the brain and readily passes through the blood brain barrier reaching CSF concentrations 25% of that found in plasma. However, whether long-term administration of 2-CDA can lead to any adverse effects in patients or animal models is not yet clearly known.

**Methodology:**

Here we show that exposure of 2-CDA to CHO cells stably expressing wild-type APP751 increased generation and secretion of amyloid β peptide (Aβ) in to the conditioned medium. Interestingly, increased Aβ levels were noticed even at non-toxic concentrations of 2-CDA. Remarkably, chronic treatment of APdE9 mice, a model of Alzheimer's disease with 2-CDA for 60 days increased amyloid plaque burden by more than 1-fold. Increased Aβ generation appears to result from increased turnover of APP as revealed by cycloheximide-chase experiments. Additionally, surface labeling of APP with biotin and immunoprecipitation of surface labeled proteins with anti-biotin antibody also indicated increased APP at the cell surface in 2-CDA treated cells compared to controls. Increased turnover of APP by 2-CDA in turn might be a consequence of decreased protein levels of PIN 1, which is known to regulate *cis*-*trans* isomerization and phosphorylation of APP. Most importantly, like many other oncology drugs, 2-CDA administration led to significant delay in acquiring a reward-based learning task in a T maze paradigm.

**Conclusions:**

Taken together, these data provide compelling evidence for the first time that chronic 2-CDA administration can increase amyloidogenic processing of APP leading to robustly increased plaque burden which may be responsible for the observed deficits in learning skills. Thus chronic treatment of mice with 2-CDA can have deleterious effects *in vivo.*

## Introduction

Cladribine or leustatin (2-chloro-2′-deoxyadenosine or 2-CDA) is a synthetic chlorinated analog of deoxyadenosine which interferes with DNA repair resulting in strand breaks and apoptosis [1], [2]. The compound was developed based on the finding that an inherited deficiency of adenosine deaminase enzyme leads to selective lymphopenia and an immunodeficiency disease in humans [3], [4] as a result of accumulation of triphosphorylated nucleotides in lymphocytes [2]. Adenosine deaminase is unable to cleave 2-CDA because of substitution of hydrogen with a chlorine atom at position 2 of the purine ring of deoxyadenosine. Once 2-CDA is taken up by cells through specific nucleotide transporters, it is phosphorylated by deoxycytidine kinase (DCK) through multiple steps resulting finally in 2-chlorodeoxyadenosine 5′-triphosphate (2-CdATP), which is the active form [5]. Since both activated and resting lymphocytes have high levels of DCK, 2-CDA accumulates at high levels within lymphocytes making them more vulnerable to cytotoxic effects [6]. Results from RNA profiling studies also suggest that DCK levels are high in T cells, B cells and dendritic cells but low in nonhematologic cell types such as liver, brain, lung, heart, skin, kidney and germ cells [7], [8], which may have an advantage of reduced adverse events related to these organs for 2-CDA. The mechanism by which 2-CdATP induces cell death appears to be through DNA double strand breaks, failure to repair them and disruption of DNA synthesis by inhibition of key enzymes involved in DNA and RNA synthesis and repair such as DNA polymerase and ribonucleotide reductase [9]–[11]. 2-CDA can induce cell death at clinically relevant concentrations through apoptosis by depleting intracellular pools of nicotinamide adenine dinucleotide and adenosine triphosphate [12]. In more recent years, autophagy has been suggested as the predominant method of cell death induced by 2-CDA [13].

Because 2-CDA can selectively deplete T and B lymphocytes, a parenteral formulation of 2-CDA got FDA approval in 1980's for use in hematological disorders such as hairy cell leukemia [14], [15] as well as chronic lymphocytic leukemia and non-Hodgkin's lymphoma [16]–[18]. In addition, 2-CDA exhibits various other effects on immune system and therefore it was explored for therapeutic utility in autoimmune disorders such as glomerulonephritis associated with systemic lupus erythematosus, rheumatoid arthritis and refractory celiac disease [19]–[21]. More importantly, as activated lymphocytes are believed to be responsible for inflammatory myelin damage in multiple sclerosis (MS), 2-CDA mediated down regulation of lymphocyte levels were thought to be beneficial to MS patients. In fact, the safety, efficacy and tolerability of parenteral 2-CDA administration in MS patients were demonstrated in 3 randomized, double-blind and placebo-controlled clinical trials using both clinical outcomes and MRI measures [22]–[24]. Subsequently, the efficacy of oral form of 2-CDA in patients with relapsing-remitting multiple sclerosis (RRMS) was also demonstrated in what is now called as CLARITY (Cladribine Tablets Treating Multiple Sclerosis Orally) study [25]. The promising results from CLARITY trial led to the first approval of oral formulation of 2-CDA for MS in 2010 in Russia under the trade name, Movectrol R [26], [27] and almost immediately in Australia, though the approval limits its use for two years in the first instance [26], [28], [29]. But European agency EMA rejected the use of 2-CDA for RRMS in the European Union citing safety concerns [30]. The United States FDA also halted approval of oral 2-CDA for RRMS until more data on safety for long-term use becomes available [31]. Thus, whether long-term use of 2-CDA is associated with any adverse effects is an important question that needs to be answered.

It is quite well known that there is cognitive dysfunction among cancer survivors also known as “chemo brain” [32], but whether chemotherapy is responsible for the observed cognitive deficits is not clear [33], though many studies have reported positive correlation between cancer chemotherapy and cognitive impairment (for review, [34]). It is also not known whether chronic treatment with 2-CDA in patients with MS and hematological cancers similarly leads to cognitive impairment. 2-CDA easily penetrates the blood brain barrier (BBB) reaching concentrations of approximately 25% of that of the plasma levels in patients with malignancies [35]. Although rare, life threatening and fatal neurotoxicity have been reported for 2-CDA when used at higher than recommended doses [36]. In mice, high doses of 2-CDA is both teratogenic and lethal to embryos, although there is no evidence of teratogenicity in humans [37]. Also there are studies showing axonal degeneration and secondary demyelination in patients with myeloid leukemia treated with 2-CDA [38] and occasionally sensorimotor polyneuropathy complicated by paraplegia and progressive coma [39]. Thus, so far there are no studies addressing whether long-term treatment with 2-CDA has any adverse effects especially on neurotoxicity in the central nervous system (CNS) and the associated cognitive function. Since the scope of clinical uses of 2-CDA is now beyond the hematological disorders and is being extended to treat CNS disorders [26]–[29], this information becomes even more critical at this juncture.

Here we show for the first time that 2-CDA exposed to CHO cells significantly increased the secretion of amyloid β peptide (Aβ) and c-terminal fragments (CTFs) in cell cultures. Also, chronic treatment with 2-CDA for 60 days in a mouse model of Alzheimer's disease, increased amyloid plaque burden by more than one-fold at six months of age. More importantly, these alterations led to significant deficits in learning skills in the food-reward based T maze paradigm.

## Methods

### Chemicals and antibodies

The FDA-approved oncology drug set II with 89 compounds at 20 mM concentrations in DMSO were obtained from NCI/NIH DTP open chemical repository [40]. 2-Chloro-2′-deoxyadenosine or 2-CDA or Cladribine (cat# C4438), thioflavin S (cat #T1892), EGTA (cat # E4378), paraformaldehyde (cat # P6148) and glutaraldehyde (cat # G-7776) were purchased from Sigma-Aldrich (St. Louis, USA). EZ-Link Sulfo-NHS-LC-Biotin was from Pierce. The polyclonal antibody CT15 (against C-terminal 15 residues of APP) and the polyclonal antibody, 63G (against mid region of APP) have been described previously (40, 41). The monoclonal antibody 6E10 (cat # SIG-39300, recognizing 1–17 of Aβ sequence) was obtained from Covance Research (Denver, USA). Polyclonal anti-sAPPβ-WT antibody (cat # 18957) was purchased from IBL Co. Ltd (Gunma, Japan). Polyclonal PIN1 antibody (cat # 3722) was purchased from Cell Signaling (Danvers, MA, USA). Mouse monoclonal antibody against beta-actin (cat # A00702) was purchased from Genscript USA Inc. (Piscataway, NJ, USA). Secondary antibodies such as peroxidase-conjugated AffiniPure goat anti-mouse (Code # 115-035-146) and ant-rabbit (code # 111-035-144) IgGs were purchased from Jackson ImmunoResearch Laboratories (West Grove, PA, USA). Anti-mouse IgG and anti-rabbit IgG-agarose beads were from American Qualex International (San Clemente, CA, USA).

### Quantitation of Aβ, CTF and sAPPβ levels in 7WD10 cells

The methods initially involved generation and characterization of CHO cells stably expressing APP751wt (7WD10 cells) for the secretion of Aβ in to the conditioned medium (CM) as described previously [41], [42]. For the immunoprecipitation of Aβ, 7WD10 cells were grown in 6-well plates and treated with 2-CDA at the final concentrations of 0, 0.1, 1.0, 5.0, 10.0 and 20.0 µM in duplicate wells. After 48 hours of drug exposure, the CM was collected, centrifuged to remove cell debris and immunoprecipitated overnight using a monoclonal Ab9 antibody (recognizes 1–16 amino acids of Aβ) to pull-down Aβ. Aβ was separated on NuPAGE 4–12% bis-tris gels and detected by immunoblots using a mixture of 6E10/82E1 antibodies as described in our published papers [40], [41]. The CM was also immunoblotted using 10% bis-acrylamide gels to detect sAPPα (6E10), sAPPβ (anti-sAPPβ-wt Rabbit IgG from IBL America ltd) and sAPPtotal (63G) using indicated antibodies. To detect APP and CTFs (CT15 antibody), the cells were lysed using lysis buffer (1% Nonidet P-40) with complete protease inhibitor mix (Sigma).). Equal amounts of proteins were loaded into each well and subjected to electrophoresis. The proteins were then transferred onto PVDF membranes, blocked with 5% milk and incubated overnight with primary antibodies followed by one hour incubation with HRP-conjugated secondary antibodies such as monoclonal mouse anti-Goat IgG light chain or monoclonal mouse anti-Rabbit IgG light chain. The protein signals were detected using Super Signal West Pico Chemiluminescent substrate (Pierce, USA). Quantification of Western blot signals was done using imageJ software.

### APP surface biotinylation and turn-over experiments

Confluent 7WD10 cells in duplicate 6-well plates were treated with 2-CDA at 10.0 µM concentration and after 24 h, washed 2 times with cold PBS and incubated with 2.0 mg/ml sulfo-NHS-LC-biotin in PBS, pH 8.0 under ultra-low shaking on ice in the cold room. After I h incubation, cells were washed three times with PBS and lysates prepared using 1% Nonidet P-40 with complete protease mix. Biotinylated proteins were pulled-down by immunoprecipitation with anti-biotin antibody plus anti-mouse agarose beads. The samples were subjected to SDS-PAGE and APP was detected with CT15 antibody. For cycloheximide experiments, 7WD10 cells were incubated with cycloheximide at the concentration of 100 mg/ml in PBS at 37°C. Control cells which did not receive cycloheximide and cycloheximide treated cells were lysed at 0, 15, 30, 60, and 120 minutes. The lysates were immunoblotted and APP was detected with CT15 antibody.

### Cytotoxicity assays

Since oncology drugs are basically designed to kill cells, we wanted to identify the minimal concentrations of 2-CDA necessary for cytotoxicity. Also, to understand whether 2-CDA toxicity is cell-type dependent, we studied cytotoxicity in both neuronal and non-neuronal cell lines. Human embryonic kidney (HEK) cells and Neuro-2A (N2A) cells were incubated with 2-CDA at the final concentrations of 0, 0.1, 1.0, 5.0, 10.0, 80.0 and 240.0 µM for 24 h. The generation of N2A and HEK cells has been described in our previous publications [41], [42]. To determine cell viability, first we used the calorimetric MTT (3-(4,5-dimethylthiazol-2-yl)-2,5-diphenyltetrazolium bromide) metabolic activity assay using cell growth determination kit (cat # CGD-1, Sigma Aldrich, St. Louis, USA) according to the manufacturer's instructions. Briefly, the supernatant was removed, cells were washed twice with PBS and 20 ul of MTT solution (5 mg/ml in PBS) plus 100 ul of medium were added. After 4 h incubation at 37° C, the resulting formazan crystals were dissolved in 100 ul of dimethyl sulfoxide and the absorbance was read at 570 nm within an hour using a spectrophotometer (Bio-Rad). Cells treated with medium only formed controls.

We also measured cell viability using *in vitro* toxicology assay kit based on lactic dehydrogenase (LDH) (cat # TOX7, Sigma Aldrich, St. Louis, USA). The assay is based on reduction of nicotinamide adenine dinucleotide (NAD) by LDH enzyme into NADH which converts tetrazolium dye in to a colored compound that can be quantitated spectrophotometrically. The procedure briefly is as follows. After 24 h incubation with different concentrations of 2-CDA, 50 ul of LDH assay lysis solution was added in to each well and further incubated at 37°C for 45 minutes. The assay mixture was freshly prepared each time by adding equal volumes of substrate, dye and cofactor. A 50 ul of LDH assay mixture was added to each of the 50 ul aliquots of the test medium, the plates were sealed with aluminum foil to protect from light and incubated for 30 minutes at 37°C. The reaction was terminated by adding 10 ul of 1 N HCl and the absorbance was measured at a wavelength of 490 nm.

### Staining for amyloid plaques

We used APdE9 mice that overexpress APP Swedish and PS1 ΔE9 mutations as a mouse model of Alzheimer's disease. All animal procedures were carried out in strict accordance with the National Institute of Health's ‘Guide for the Care and Use of Animals’ and approved by the Torrey Pines Institute's Animal Care and Use Committee (IACUC, approval # TPI-11-03). Mice were administered daily by intraperitoneal injections of 2-CDA at 0.5 mg/kg body weight starting from four months of age until six months of age for 60 days. Control mice received vehicle injections only for the same period of time. At the end of treatment period and after the behavioral tests, mice were anesthetized by isoflurane and perfused using a mixture of 4% paraformaldehyde (PFA) and 0.02% glutaraldehyde in phosphate buffered saline (PBS). After 72 h the brains were dehydrated using 10%, 20%, and 30% sucrose gradients. Brains were frozen in OCT and coronal sections of 16 µm thickness was cut by cryostat at −20°C and transferred on to super frost slides. Antigen retrieval was carried out by placing slides in citrate buffer (10 mM, pH 6.0) at 95 −100°C for 5 min. The slides were then rinsed with distilled water 2x for 5 min. The slides were then immersed in 1% Thioflavin S solution prepared in water for 5 min and then differentiated in 70% ethanol for 5 min, rinsed again in water 2x for 5-min and cover-slipped with Sure mount (EMS) mounting media and placed at 4°C until they were imaged. Images were captured using a Zeiss Examiner D1 microscope. All images were acquired at the same exposure and were automatically aligned using the stitching tool in the Axiovision LE software. Once acquired, all images were opened in image J and normalized, the threshold was set for each image using the histogram mean at the same standard deviation. Each image was adjusted to the threshold, and set the scale in pixels. The parameters measured include the area, integrated density, perimeter, and feret's diameter for each plaque. To help eliminate background the particle size pixel was set at 30 –infinity pixel. To quantify plaques the brain level of sections cut were fixed for all mice at the region of motor cortex and hippocampus. A fixed thickness of 16 um coronal sections at regular intervals was maintained in all animals. The amyloid plaques were quantified from throughout the sections from five sections per mouse and mean values were generated for each mouse. Pictures were montaged and for quantification by image J software, the color images were converted in to HSV format and 8 bit channels. Plaques were quantified in an unbiased manner by an investigator blind to the treatment nature of the samples. Plaque burden was calculated based on the percentage of the area covered by the plaques over the total area of the brain sections. The data is expressed as percent change from the controls.

### Behavioral memory test

We tested spatial learning and memory skills in mice using T maze. In this paradigm mice are initially trained to learn a given task and then mice are subjected to the probe test to check their ability to retain the learnt task. We used a custom-made T maze with a design slightly modified from that of Deacon and Rawlins, 2006 [43]. The maze was made of 12 mm thick white acryl material. The vertical arm was 12 inches in length with an additional start box at the front with measurements of 4.5 inches on all sides. The horizontal arm consisted of the left and right arms, each was 12 inches long with 2.5 inch diameters. Three Guillotine doors were also placed, one between the start box and the vertical arm at a distance of 2 inches and the other two on either side of the left and right arms at a distance of 1.5 inches from the point where the vertical arm meets the horizontal arm. The entire T maze was in turn placed within the platform which is also made up of acryl material. The platform was necessary to place all the electronic circuits and electrical switch controls for light emitting diodes (LEDs) placed on each side of the right and left arms to provide enough light for visibility.

The mice were fed with *ad libitum* food and water all the time. The food is the irradiated global rodent chow from Harlan. Only during the experimental periods mice were deprived of food for about 12–14 h per day. As a reward, a small food pellet was placed on a small petri dish in one of the arms called bait arm. The pellet was placed at the bait arm each time just before start of the session. All experiments were carried out during the day with lights on at the same time of the day to minimize circadian rhythm based alterations in the mice behavior. The training session was started on day 48 of drug injection until day 52 for a total of five days. Each mouse was initially habituated to the environment by placing them in the T maze for five minutes, with all the Guillotine doors kept open so that each arm was freely accessible with food as reward at the end of the two goal arms. To begin the training session, each mouse was placed for 30 sec in the start box and then the door at the start box was open and the time was noted. The trials measured the latency, i.e., the time taken from the start box to reach the baited arm. The probe test was conducted after a gap of seven days on the day 59 of drug injections. The probe test consisted of at least four trials for the mice to correctly reach the baited arm. The time taken by the mice to correctly reach and find the reward was noted and counted as latency. Mice received drug injections throughout the entire period of behavioral test until day 60 when they were euthanized and perfused for immunohistochemical studies.

### Statistical analysis

Signal intensities of immunoblots of the samples treated with 2-CDA in CHO cells as well as plaque burden in the mouse brain were quantified using publicly available Java-based Image J software developed at the National Institute of Health. Data were analyzed by Student's t test or by analysis of variance (ANOVA) followed by post-hoc test using Instat3 software (GraphPad Software, San Diego, CA, USA). We used both one-tailed and two-tailed p value assuming populations may have different standard errors. The data were considered significant only if the p<0.05, * indicates p<0.05, **, p<0.01 and ***, p<0.001.

## Results

### 2-CDA significantly increases Aβ levels in CHO cells

**Figure 1:**
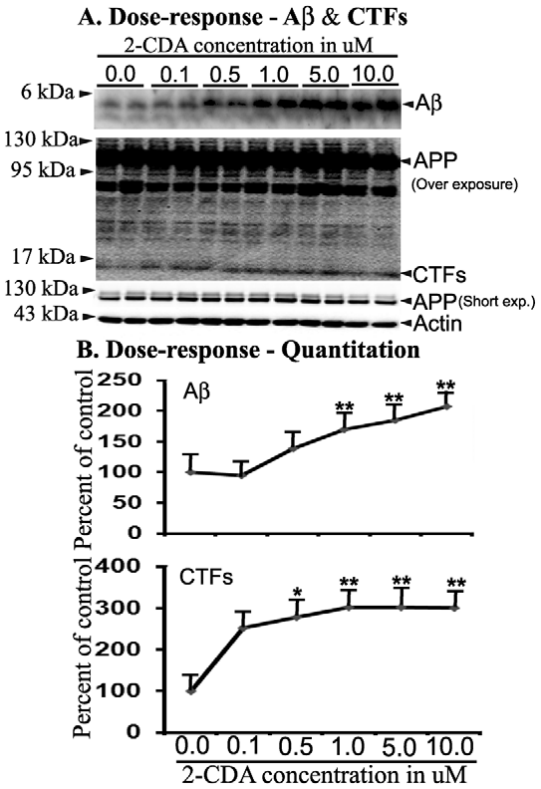
2-CDA increases Aβ and CTF levels in CHO cells stably expressing WT APP751 (7WD10 cells). (A) 7WD10 cells were exposed to different concentrations of 2-CDA for 48 h and the conditioned medium was immunoprecipitated for Aβ and detected by immunoblots using NuPAGE 4–12% bis-tris gel. The lysates were used to detect c-terminal fragments (CTFs) using NuPAGE gels. APP as well as actin as a loading control were separated using 10% bis-acrylamide gels. (B) Quantitation by image J revealed increased levels of Aβ at 1.0 µM (69% (p<0.01), 5.0 µM (84%, p<0.01) and 10.0 µM (107%, p<0.01) compared to controls. Values are expressed as percentage change from controls. CTF levels were also significantly increased at 0.5 µM (278%, p<0.05), 1.0 µM (302%, p<0.01), 5.0 µM (302%, p<0.01) and 10.0 µM (301%, p<0.01) compared to controls. For all samples, n = 4. *, p<0.05, **, p<0.01, versus control by analysis of variance (ANOVA) followed by post-hoc test by Dunnett multiple comparisons.

Anecdotal observations in nursing homes that Alzheimer's disease (AD) patients were less likely to have diagnosed with cancer and vice versa strongly suggest inverse relationship between cancer and AD [44]. These evidences led us to strongly believe that oncology drugs might be helpful in AD. Therefore we screened a library of all the so far FDA-approved oncology drugs of total 89 compounds obtained from NCI/NIH for their effect on Aβ levels. Although cumbersome, we used immunoprecipitations/blotting to screen all the drugs to identify those drugs that alter Aβ levels. While some drugs reduced the levels of Aβ, which will be presented elsewhere, 2-CDA robustly increased Aβ levels in CHO cells. Indeed, dose-response experiments confirmed 2-CDA induced increased Aβ levels and also other amyloid precursor protein (APP) metabolites. To test the effect of different concentrations of 2-CDA, CHO cells stably expressing APP751 WT were treated for 48 h and the conditioned medium were immunoprecipitated with Ab9 antibody which recognizes an epitope within 1–16 amino acids of Aβ peptide. This was followed by Western blot detection of Aβ using 6E10/82E1 mixture of antibodies which we have previously used for consistent detection of total Aβ species. Exposure of 7WD10 cells to 2-CDA increased the secretion of Aβ. Increasing the concentration of 2-CDA revealed a dose-dependent increase in Aβ levels starting from 1.0 µM (69%, p<0.01), 5.0 µM (85%, p<0.01 and 10.0 µM (107%, p<0.01), without altering the level of holoprotein at any concentration tested (Figure 1A and B, upper panels). These results demonstrate that 2-CDA stimulates β/γ-secretase mediated APP cleavage.

### 2-CDA significantly increases c-terminal fragment (CTF) levels in CHO cells

While we used the conditioned medium for quantifying Aβ levels, the lysates from the same cells treated with different concentrations of 2-CDA were used to quantify the levels of CTFs derived from APP by immunoblotting using CT15 antibody. Results showed that 2-CDA increased CTF levels to 253% at 0.1 µM, 278% (p<0.05) at 0.5 µM, 302% (p<0.01) at 1.0 µM, 302% (p<0.01) at 5.0 µM and 301% (p<0.01) at 10.0 µM concentrations compared to DMSO treated controls (Figure 1A and B, lower panels). Thus there was more than two-fold increase in the levels of CTFs which were all statistically significant except the lowest concentration of 0.1 µM. The level of APP holoprotein which was also recognized by the CT15 antibody was not changed at any of the 2-CDA concentrations. The increased CTF levels is consistent with increased Aβ levels by 2-CDA and indicate that 2-CDA increases amyloidogenic processing of APP in CHO cells.

### 2-CDA significantly increases levels of sAPPβ but not sAPPα or sAPPtotal

**Figure 2:**
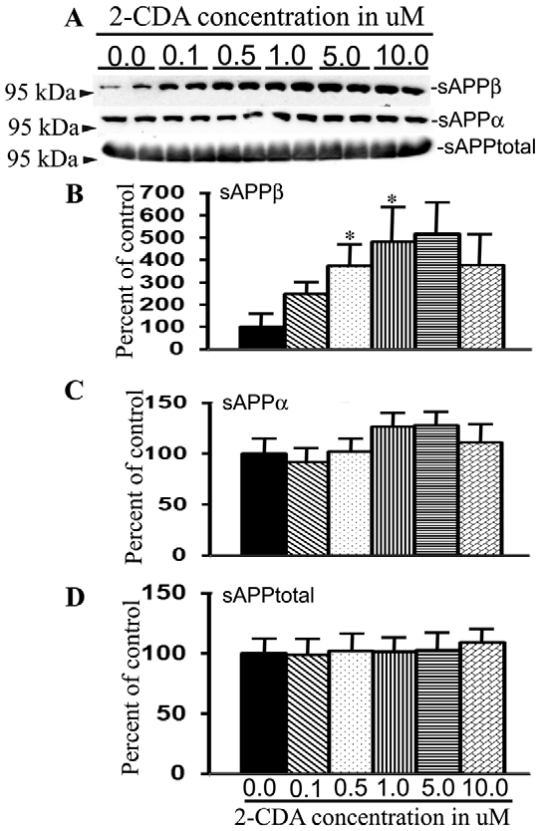
2-CDA increases sAPPβ levels but not sAPPα or sAPPtotal. (A) 7WD10 cells were treated with different concentrations of 2-CDA as indicated and after 48 h the conditioned medium was subjected to immunoblotting to detect different species of sAPPs. (B) sAPPβ levels were increased to 250% at 0.1 µM, 375% (p<0.05) at 0.5 µM, 484% (p<0.05) at 1.0 µM, 518% at 5.0 µM and 378% at 10.0 µM compared to untreated cells. (C) sAPPα levels were not significantly altered in 2-CDA treated cells versus controls at any of the concentrations. (D) Levels of sAPPtotal were also not altered in 2-CDA treated cells. For all samples, n = 4. *, p<0.05 versus controls by repeated measures ANOVA followed by Dunnett multiple comparison test.

To get an overall picture of APP metabolism under 2-CDA treatment, we further quantified the levels of secreted large extracellular N-terminal domain truncated at the α-site (sAPPα) or at β-site (sAPPβ) as well as levels of sAPPtotal from the same conditioned medium used for Aβ quantification. More than two-fold increase was noted for sAPPβ levels at various concentrations of 2-CDA treated for 48 h, though we did not notice strict dose-dependent increase in sAPPβ levels detected using a polyclonal anti-sAPPβ-WT antibody, which specifically detects sAPPβ protein from wild-type APP. The secretion of sAPPβ was 250% at 0.1 µM, 375% (p<0.05) at 0.5 µM, 484% at 1.0 µM (p<0.05), 518% at 5.0 µM and 378% at 10.0 µM over the basal levels (Figure 2A upper panel and 2B). The maximal increase was noted at 5.0 µM concentration to 518% but for some unknown reason the levels of sAPPβ decreased to 378% at 10.0 µM. On the other hand there were no significant alterations in the levels of both sAPPα (Figure 2A middle panel and 2C) and sAPPtotal (Figure 2A lower panel and 2D) at any of the 2-CDA concentrations tested. The sAPPα levels were detected with 6E10 antibody which recognizes an epitope within the 1–17 of the Aβ domain of APP. sAPPtotal was detected by 63G antibody whose epitope lies within the mid region of APP. APP is predominantly processed by α-secretase generating sAPPα. The quantitative difference between sAPPα to sAPPβ is almost 1000 fold. Even if sAPPβ levels are increased by 4-folds as observed in the present study, its contribution to sAPPtotal is still not high. The increase in sAPPα levels were however not significant. Thus, 2-CDA treatment increased sAPPβ levels, but the levels of sAPPtotal remained unchanged. Taken together, increased levels of Aβ, CTFs and sAPPβ suggest that 2-CDA may increase amyloidogenic processing of APP through β-secretase pathway.

### 2-CDA increases turnover of APP

**Figure 3:**
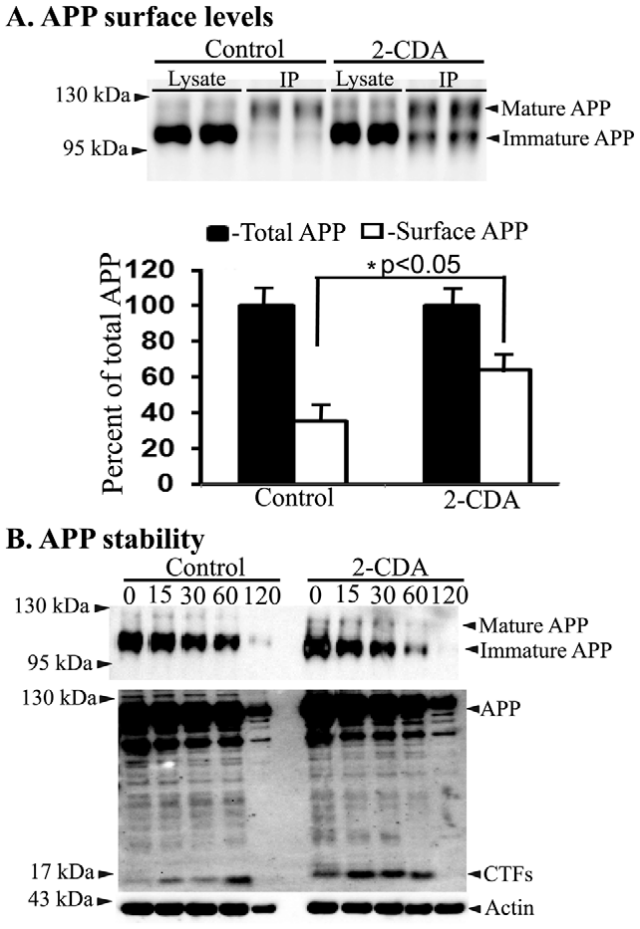
2-CDA increases surface levels of APP and its turnover. (A) 7WD10 cells were treated with 2-CDA at 1.0 µM concentration followed by cycloheximide chase at indicated time points in 2-CDA treated and untreated cells (upper panel). The lower panel is the overexposed blot to show accumulation of CTF levels in 2-CDA treated cells versus controls at different chase times. (B) Surface biotinylation and biotin immunoprecipitation showed significantly increased surface levels of APP in 2-CDA treated 7WD10 cells compared to untreated cells. (C) Quantitation of immunoblot signals by imageJ revealed significant differences (p<0.05) in surface levels of APP but not in the levels of total APP in 2-CDA treated cells versus controls. Values are expressed as percentage change in surface levels of APP in control versus 2-CDA treated cells. For all samples, n = 4. *, p<0.05 versus control by student t test.

To examine whether increased amyloidogenic processing of APP is due to increased turn-over of APP, we did cycloheximide chase experiments. Confluent 7WD10 cells in 6-well plates treated with or without 2-CDA were incubated with 100 mg/ml of cycloheximide to inhibit *de novo* synthesis of APP. This was followed by quantitation of steady state levels of APP holoprotein by immunoblots using CT15 antibody at different time points of up to 2 h based on our previous experience that the calculated half-life of APP was about 1 h [41]. The results showed that by one hour there was less than half the level of APP in 2-CDA treated cells compared to control cells. By two hours, while there was still a measurable quantity of APP in the control cells, there was no trace of APP in 2-CDA treated cells suggesting that 2-CDA increases the turnover of APP (Figure 3A). The increased turnover of APP is also in line with increased Aβ, CTF and sAPPβ levels in 2-CDA treated 7WD10 cells. Also, the levels of both mature and immature APP at the cell surface were increased by 2-CDA in our APP surface biotinylation experiments (Figure 3B & C). The increased surface levels of APP by 2-CDA might lead to increased endocytosis which has been suggested to be necessary for cleavage of wild-type APP by secretases and Aβ production [45], [46].

### Chronic 2-CDA administration increases plaque burden in mice

**Figure 4:**
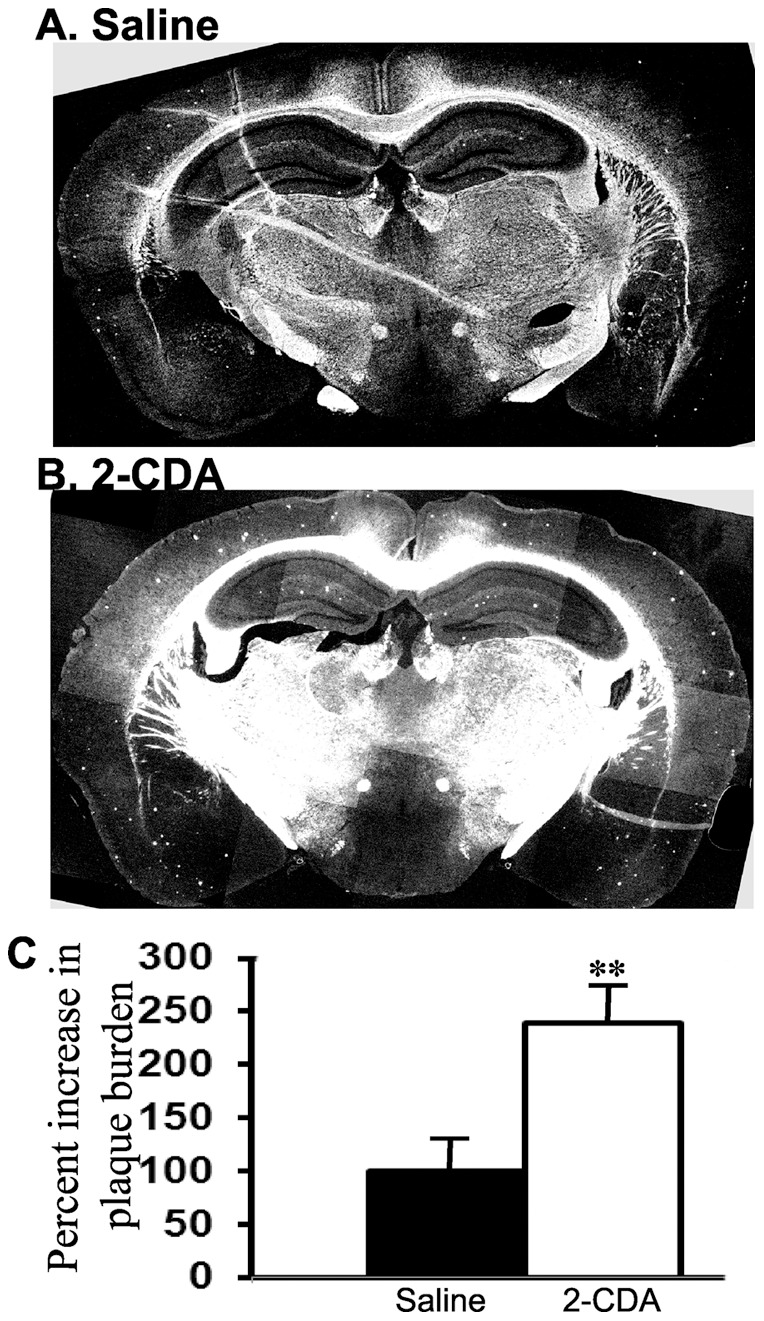
Chronic 2-CDA administration increases amyloid plaque burden in mice. (A) APdE9 mice were treated daily by intraperitoneal injections with 2-CDA starting from four months of age to six months of age for 60 days. Amyloid plaques were stained by Thioflavin S in coronal brain sections. A representative section with amyloid plaques in untreated mice at six months of age is shown in A. (B) a representative section of brain showing increased amyloid plaques in mice treated with 2-CDA is shown. (C) Quantitation of plaque burden by imageJ analysis showed increased burden to 240% (p<0.01) when compared to saline treated APdE9 mice. In each group, n = 4. **, p<0.01 in 2-CDA treated APdE9 mice versus untreated APdE9 mice by student t test.

Next, to verify whether increased amyloidogenic processing of APP by 2-CDA observed in cell cultures is translated *in vivo* into increased plaque burden, mice overexpressing both APP with Swedish mutation and PS1 with ΔE9 deletion was used as a robust mouse model for Aβ plaques (APdE9) as they develop modest amount of amyloid plaques as early as six months of age. Six mice were injected with saline and another six mice with 2-CDA chronically for 60 days by intraperitoneal injections from 4 months of age to 6 months of age. Overall plaque burden was calculated as the ratio of ‘the area occupied by plaques to the total region area’, which was clearly increased by more than one-fold (140%) in 2-CDA treated mice compared to saline treated mice (Figure 4C). Representative histology sections for each of saline or drug treated mice are shown in Figure 4A and B respectively at comparable brain level. Increased plaque burden in 2-CDA treated mice indicates that 2-CDA increases larger plaques compared to no treatment group. We observed that plaques were of so many different sizes and intensities, though there were not numerous plaques as one would expect in an older mice, since the mice were only six months old. Quantification of overall density of plaques confirmed a significant difference between the drug treated and saline treated groups of mice. The increased amyloid plaque burden following 2-CDA treatment in mice is also consistent with our interpretation of cell culture data that 2-CDA increases amyloidogenic processing of APP.

### Cytotoxicity of 2-CDA as measured by LDH release and MTT reduction

**Figure 5:**
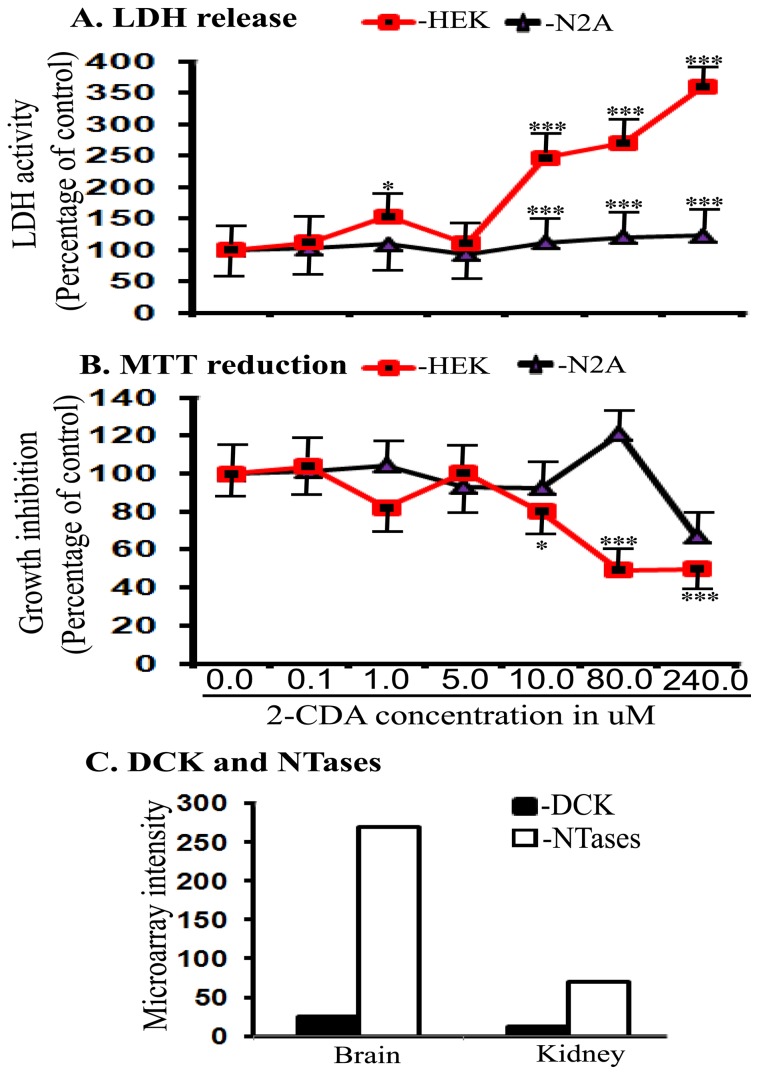
2-CDA is cytotoxic at high concentrations. (A) LDH release was measured as an indicator of cytotoxicity in HEK 293 cells and N2A cells after incubation with different concentrations of 2-CDA as indicated. Significant cytotoxicity was observed at 1.0 µM (p<0.05), 10.0 µM (p<0.001), 80.0 µM (p<0.001) and 240.0 µM (p<0.001) in HEK 293 cells. Similarly in N2A cells significant toxicity was observed at 10.0 µM (p<0.001), 80.0 µM (p<0.001) and 240.0 µM (p<0.001). (B) MTT reduction assay, however indicated significant toxicity only in HEK 293 cells at 10.0 µM (p<0.05), 80.0 µM (p<0.001) and 240.0 µM (p<0.001), but 2-CDA was nontoxic in N2A cells even at high concentrations. (C) the calculated DCK activity was almost one-fold higher in the brain compared to kidneys. But kidneys showed almost three-fold increased 5′-NTases activity compared to whole brain tissue. For all samples in A and B, n = 4. *, p<0.05, **, p<0.01, ***, p<0.001 versus controls by ANOVA followed by Tukey-Kramer multiple comparison test.

We used two dehydrogenase enzyme based reductive coloring reagent methods for the detection of cytotoxicity based on colorimetric detection since they are simple, easy to use, safe and highly reproducible. Determination of mitochondrial dehydrogenase activity by MTT reduction revealed that 2-CDA was non-toxic up to 5.0 µM concentrations in both human embryonic kidney derived 293 (HEK 293) cells and neuron-derived neuro-2A (N2A) cells (Figure 5B). However, 2-CDA incubation at 10.0 µM, 80.0 µM and 240.0 µM were highly toxic at each concentrations (p<0.001) consistently in both HEK cells and N2A cells (Figure 5B). To confirm these results in another independent assay, we used conditioned medium from the cells incubated with different concentrations of 2-CDA to quantify the amount of lactate dehydrogenase (LDH) enzyme. Similar to MTT results, LDH release was not significantly altered in both HEK 293 and N2A cells up to 5.0 µM (Figure 5A). But at 10.0 µM and higher concentrations, 2-CDA was significantly toxic in only HEK 293 cells but not in N2A cells (Figure 5A). Thus, it is interesting to note that 2-CDA were less toxic to neurons than kidney cells, which is quite contrary to the wide belief that neurons are more vulnerable to toxicity than non-neuronal cell types.

Since 2-CDA must be converted in to an active form 2-CdATP by DCK enzyme [5], we compared the levels of DCK in kidney and the brain tissues to see if the differences in the activities of DCK could account for the differential toxicity in neuron versus kidney cells. Also, since most cells also contain another enzyme, 5′-nucleotidases (5′-NTases) which balances the activity of DCK and prevent generation of 2-CdATP, we also calculated the ratios of expression of DCK mRNA to 5′-NTases mRNAs in kidney and whole brain tissues from the data available at BioGPS website [47]. As shown in Figure 5C, although DCK activity in the brain was about 1-fold more than that calculated from kidneys which is expected to generate more 2-CdATP, the activities of NTases were almost 3-fold more in the brain than kidneys which would prevent accumulation of 2-CdATP in the brain. Thus the differential activities of DCK and 5′-NTases accounts for the differences seen in cytotoxicity of 2-CDA in the neuronal cell versus kidney cells.

### Chronic 2-CDA administration increases latency in T maze learning

**Figure 6:**
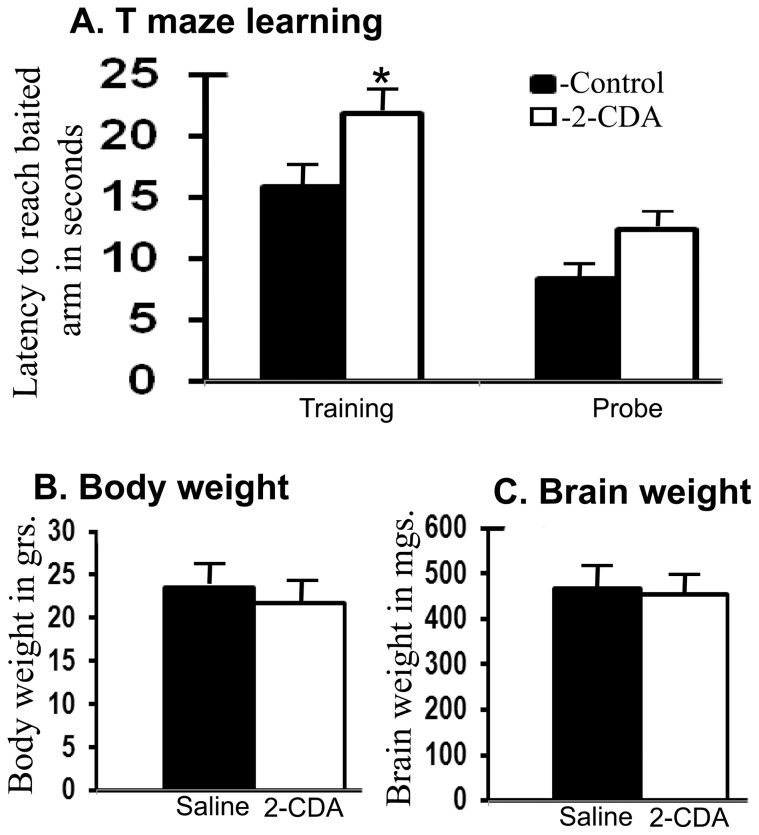
Chronic 2-CDA administration in mice delays reward-based learning in a T maze paradigm. (A) APdE9 mice treated with 2-CDA for 60 days by intraperitoneal injections from four-months of age to six months of age showed 38% (p<0.05) delay in latency in acquiring food pellet-based learning task in the T maze. However, once the task was learnt, they no longer differed from controls in their retention of memory (probe). (B) Chronic 2-CDA administration did not alter the body weights or the brain weights (C). n = 6 in each group. *, p<0.05 versus controls by student t test.

Chronic 2-CDA treatment for 60 days did not lead to significant changes in either body or brain weights, suggesting that 2-CDA does not induce gross alterations in the mice (Figure 6B and C). But there is growing evidence that many cancer drugs impact cognitive function even at clinically relevant doses. Therefore to assess whether 2-CDA induced increased amyloid plaque burden leads to behavioral learning deficits, we subjected six APdE9 mice treated with 2-CDA and another six saline treated mice in a simple T maze learning task for their spatial memory skills. It is quite apparent in Figure 6A that drug treated mice took significantly more time (latency) to find the food pellet as reinforcement at the baited arm (p<0.05) during five day training period. However, once both the groups learnt the task of reaching the baited arm, there were no significant differences in the retention of memory as judged from probe test carried out on day 7 after the training was completed.

### 2-CDA decreases PIN 1 protein levels

**Figure 7:**
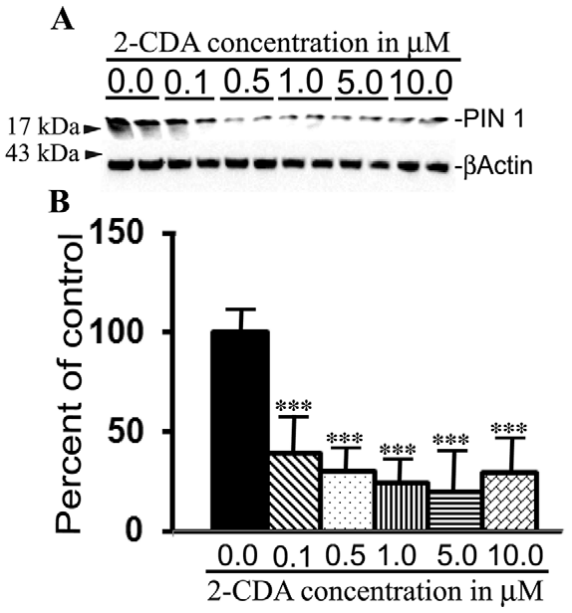
2-CDA decreases PIN 1 protein levels in 7WD10 cells. (A) Cells were treated with 2-CDA at different concentrations as indicated and after 48 h, the lysates were immunoblotted to detect PIN 1 protein and actin as a loading control. (B) ImageJ quantitation revealed significantly decreased PIN 1 protein levels at 0.1 mM (39%, p<0.001), at 0.5 µM (30%, p<0.001), 1.0 µM (24%, p<0.001), 5.0 µM (19%, p<0.001) and 10.0 µM (29%, p<0.001) compared to untreated control cells n = 4 in each group. *, p<0.05, **, p<0.01, vs. untreated controls.

Finally, to understand the possible molecular mechanism for 2-CDA-induced amyloidogenic processing of APP we quantified the levels of PIN1 protein since PIN 1 helps keep APP in proper isomerization form in a cell-cycle dependent manner which can favor amyloidogenic processing of APP. 7WD10 cells exposed to different concentrations of 2-CDA revealed highly significant reductions at 0.1 µM (39%, p<0.001), at 0.5 µM (30%, p<0.001), 1.0 µM (24%, p<0.001), 5.0 µM (19%, p<0.001) and 10.0 µM (29%, p<0.001) compared to untreated control cells (Figure 7A & B).

## Discussion

The clinical uses of 2-CDA which were initially prescribed throughout the world against hematological and lymphoid malignancies is now extended to treat patients with multiple sclerosis (MS) in at least two countries [26], [28], [29] and the approval by the regulatory authorities for use in MS patients is pending in both USA and European Union for lack of further safety data [30], [31]. In an experimental plan primarily designed to identify Aβ lowering oncology drugs, accidentally we found that 2-CDA, instead robustly increased Aβ generation. In this paper, we show that 2-CDA treatment of CHO cells stably expressing wild-type APP751 (7WD10 cells) significantly increased generation and secretion of Aβ in to the conditioned medium. The increased Aβ levels were also translated in to increased amyloid plaque burden in a mouse model of Alzheimer's disease. Interestingly, 2-CDA treatment also affected the learning skills, but once the task was learnt, they behaved like control mice in a T maze based spatial learning paradigm.

While there are few oncology drugs which were recently demonstrated to reduce Aβ generation [48], [49], 2-CDA is the first drug among about so far 89 FDA-approved oncology drugs to potentiate Alzheimer's pathology at least in a mouse model. This is the first study reporting significant potentiation of Aβ and amyloid plaques following 2-CDA administration and therefore can't be compared with other studies. Life threatening and fatal neurotoxicity of 2-CDA has been observed only when used at higher than recommended doses of 2-CDA when plasma levels reaches about 20 to 30 nM [36]. However, even at recommended doses, about 15% of patients have reported neurotoxicity which was at least partially reversible [36]. In human, 2-CDA is administered at cumulative doses of 3.5 or 5.25 mg/kg body weight, which even for the lower dose of 3.5 mg/kg in a 70 kg adult turns out to be 245 mg per person. Since the molecular weight of 2-CDA is 285.69, this calculates to be 850 µM for an adult with approximately 5 liters of blood or 170 µM/liter. But because 2-CDA has half-life of 4.2–9.2 h and oral bioavailability of 40%, the actual maximal concentration of 2-CDA reaching tissues is much lower than the calculated amount. In patients with lymphomas, after oral administration, blood concentrations of up to 10.0 µM of active 2-CDA metabolites have been reported [35]. Since 2-CDA has greater affinity to the brain reaching as much as 25% of that seen in the blood [35], high nM concentrations of 2-CDA can be expected in the brain. Our *in vivo* dose of 0.5 mg/kg in mice is little higher than the recommended dose in human when doses are extrapolated from human to mouse based on body surface area of 0.007 for mice and 1.6 for human. In an earlier study, the same dose of 0.5 mg/kg body weight did not induce Schwann cell damage but 1.0 mg/kg dose showed nuclear damage and vacuolization in Schwann cells as well as disorganization of the myelin sheath [50]. Although we did not measure peripheral neurotoxicity in our mice, our results suggest that even at 0.5 mg/kg dose which did not induce any damage to myelin sheath or Schwann cells [50], 2-CDA can significantly alter amyloidogenic processing of APP. Lack of cytotoxicity by 2-CDA at concentrations of up to 5.0 µM in both HEK and N2A cells measured by two independent assays also suggest that 2-CDA can significantly potentiate Aβ generation at non-toxic concentrations.

Increased turnover of APP by 2-CDA as judged from the present cycloheximide and APP surface labeling experiments may partially account for the increased Aβ generation. The turnover of APP in turn is known to be affected by PIN1 levels. The phosphorylation of APP Thr-668-Pro which is increased in Alzheimer's brains is regulated by PIN 1 [51] which may act as a conformational switch leading to altered APP turnover. The *cis* Thr-668-Pro conformation has been suggested to favor amyloidogenic processing whereas *trans* conformation to non-amyloidogenic processing of APP [51]. We have confirmed that PIN 1 levels are significantly reduced by 2-CDA which in turn is expected to alter APP conformation and therefore Aβ generation. This is also in line with the reports that PIN 1 protein levels are significantly reduced in the brains of patients with Alzheimer's disease [52], [53], which leads to increased generation and accumulation of Aβ peptides. Thus 2-CDA-induced increased Aβ generation may be due to decreased PIN 1 levels in the cells which might alter APP conformation and subsequently the turnover and processing of APP. In future directions, it will be interesting to study whether 2-CDA alters Aβ generation solely by influencing APP trafficking or whether 2-CDA has specific effects on the activities of α, β or γ-secretases. It is also important to understand whether 2-CDA influences interaction between APP and secretases by directly regulating PIN1 levels.

It is also crucial to note that chronic 2-CDA administration significantly delayed the learning skills in a T maze task, but the memory was intact. Increased Aβ and amyloid plaque burden which are widely known to cause such deficits might be directly responsible for the observed behavioral abnormality. A number of cancer chemotherapeutic drugs are known to cause chemo brain [32], [34] in which symptoms such as short-term memory loss, deficits in attention and word finding, neuropathy and muddled feelings have been reported. Most often, chemo brain cases are reported from breast cancer patients [54] who are treated with taxanes, cyclophosphamide and doxorubicin. Our current finding suggests that hematological and lymphoid cancer patients and in more recent years MS patients treated with 2-CDA also should be monitored for cognitive functions.

## Conclusions

In summary, our data has revealed for the first time that chronic 2-CDA administration could lead to significant delays in learning skills as a result of accumulation of Aβ in the amyloid plaques. Accumulation of amyloid plaques in turn may result from decreased PIN 1 levels in the brain which is known to regulate phosphorylation through *cis*-*trans* isomerization and turnover of APP.
